# Predialysis anemia management and outcomes following dialysis initiation: A retrospective cohort analysis

**DOI:** 10.1371/journal.pone.0203767

**Published:** 2018-09-26

**Authors:** James B. Wetmore, Suying Li, Heng Yan, Hairong Xu, Yi Peng, Marvin V. Sinsakul, Jiannong Liu, David T. Gilbertson

**Affiliations:** 1 Chronic Disease Research Group, Minneapolis Medical Research Foundation, Minneapolis, Minnesota, United States of America; 2 Division of Nephrology, Hennepin County Medical Center, University of Minnesota, Minneapolis, Minnesota, United States of America; 3 AstraZeneca, Gaithersburg, Maryland, United States of America; Istituto Di Ricerche Farmacologiche Mario Negri, ITALY

## Abstract

Whether and how anemia treatment with erythropoiesis stimulating agents (ESAs) before hemodialysis initiation may be associated with lower mortality after dialysis initiation is unknown. We compared all-cause and cardiovascular mortality in two groups of patients who experienced distinct anemia treatment patterns with ESAs before and after hemodialysis initiation. This retrospective cohort analysis included patients initiating hemodialysis April 1, 2012-June 30, 2013, identified from United States Renal Data System end-stage renal disease (ESRD) and pre-ESRD files. Patients treated with ESAs before and after hemodialysis initiation who maintained Hb ≥ 9.0 g/dL throughout (comparator group, *n* = 3662) were compared with patients with Hb < 9.0 g/dL before hemodialysis initiation (with or without ESAs) whose levels increased with ESAs after hemodialysis initiation (referent group, *n* = 4461). Cox proportional hazards models were used to calculate the hazard ratio of all-cause and cardiovascular mortality after hemodialysis initiation. Of 20,454 patients, 4855 (23.7%) had Hb < 9.0 g/dL upon hemodialysis initiation; of these 4855, 26.6% received ESAs before initiation. Comparator group Hb levels increased from 8.2 ± 0.8 mg/dL upon initiation to 10.9 ± 1.2 with ESAs afterward. Comparator patients were more likely than referent patients to be younger (76.3 ± 6.7 versus 77.2 ± 6.9 years), male (51.5% versus 49.8%), and black (24.6% versus 18.6%). Risk of all-cause mortality was lower for the comparator group versus the referent group at 3 (HR 0.83, 95% CI 0.68–1.00, *P* = 0.052), 6 (0.86, 0.74–1.00, *P* = 0.047), and 12 (0.88, 0.78–0.99, *P* = 0.036) months. The pattern was similar for cardiovascular mortality. Hb ≥ 9.0 with ESAs before and after hemodialysis initiation was generally associated with lower post-initiation all-cause and cardiovascular mortality compared with predialysis Hb < 9.0 g/dL in patients whose Hb levels subsequently improved with ESAs after hemodialysis initiation.

## Introduction

Anemia management is a cornerstone of care for patients with advanced chronic kidney disease (CKD). In the setting of maintenance hemodialysis, treatment with erythropoiesis-stimulating agents (ESAs) is ubiquitous [1–4]. However, anemia is also common in predialysis patients with advanced CKD [5,6]. Such patients are typically treated in outpatient office settings, where protocols for administering ESAs may be used less often and with less rigor than in dialysis units. Faced with numerous CKD-related competing priorities in medically complex patients, physicians may not be afforded the opportunity to optimally manage anemia.

Anemia is associated with mortality in patients receiving maintenance dialysis [7]. The reasons are not entirely clear, as both anemia itself and the factors contributing to it, such as inflammation, may independently contribute to mortality risk [8,9]. Regardless, the months following hemodialysis initiation are characterized by high mortality rates [10] and by concerted efforts to increase low Hb levels.

Whether and how treatment of anemia in the setting of advanced predialysis CKD might confer benefits observable after dialysis initiation is unknown. To investigate one aspect of this question, we performed a retrospective cohort analysis of patients initiating maintenance hemodialysis. We specifically sought to contrast all-cause and cardiovascular mortality in patients who were receiving ESAs and who attained specific Hb thresholds (e.g., 9.0 g/dL) before hemodialysis initiation with outcomes in patients who did not attain specific Hb thresholds (whether treated with ESAs or not) but whose Hb levels increased while receiving ESAs after hemodialysis initiation. We hypothesized that attainment of specific predialysis anemia targets with ESAs in patients who required anemia management would be associated with lower risk of mortality following hemodialysis initiation compared with pronounced correction of anemia with ESAs only after hemodialysis initiation.

## Materials and methods

### Data sources

This study used the 2012–2013 United States Renal Data System end-stage renal disease (ESRD) database, which contains information from the ESRD Medical Evidence Report and Death Notification form, the Medicare enrollment file, and the standard analytic files claims. For patients on Medicare at ESRD onset, pre-ESRD medical claims are included in the ESRD database for up to 2 years. The ESRD clinical CROWNWeb data were also used to derive patient Hb levels. We applied to and received approval from the Human Subjects Research Committee of the Hennepin County Medical Center/Hennepin Healthcare System, Inc.

### Study design and sample

This was a retrospective cohort analysis of patients who initiated maintenance hemodialysis. The study sample consisted of US patients who initiated hemodialysis between April 1, 2012, and June 30, 2013. We deliberately sought to study a period after introduction of the CMS revised Prospective Payment System (January 2011) and the Food and Drug Administration’s ESA label change (June 2011). The April 2012 starting date was chosen because CMS began requiring the reporting of Hb levels for all dialysis patients on that date.

To be eligible, patients were required to be continuously insured by Medicare Parts A and B during the 6-month baseline period (described below); to be alive on hemodialysis on the first day of the follow-up period (which began 90 days after hemodialysis initiation); to have at least one Hb measurement in the month of dialysis initiation or a Hb value recorded on the Medical Evidence Report upon initiation; to have at least two Hb measurements in the 3 months immediately after hemodialysis initiation; and to be aged 66 years or older at initiation (to permit ascertainment of Medicare claims in the pre-dialysis period). Patients who underwent blood transfusion during the baseline period were excluded.

The study timeline established a 6-month baseline period consisting of the 3 months immediately before hemodialysis initiation and the first 3 months afterward to define ESA use both before and after hemodialysis initiation, Hb levels upon initiation and thereafter, and demographic characteristics and comorbid conditions. The follow-up period used to define the outcomes began on day 91 and ended at the earliest of death, end of Medicare coverage, loss of follow-up, modality change (e.g., to peritoneal dialysis) plus 30 days, or recovery of renal function.

### Exposure

To assess the patterns in Hb levels as required by the study design, we assessed Hb levels at two time-points. The first was designed to be as close to hemodialysis initiation as possible (to ostensibly reflect primarily predialysis anemia management); levels were determined using the decision rule described in S1 Text. The second was designed to be as close to end of the baseline period (i.e., as close to the beginning of the follow-up period) as possible. In successive and parallel analyses, we used two Hb thresholds to define anemia: < 9.0 g/dL and < 10.0 g/dL. Use of ESAs after hemodialysis initiation was determined from ESRD monthly dialysis claims. Predialysis ESA use was defined from three data sources as described in the S1 Text. Relevant procedure and drug codes appear in S1 Table.

**Fig 1:**
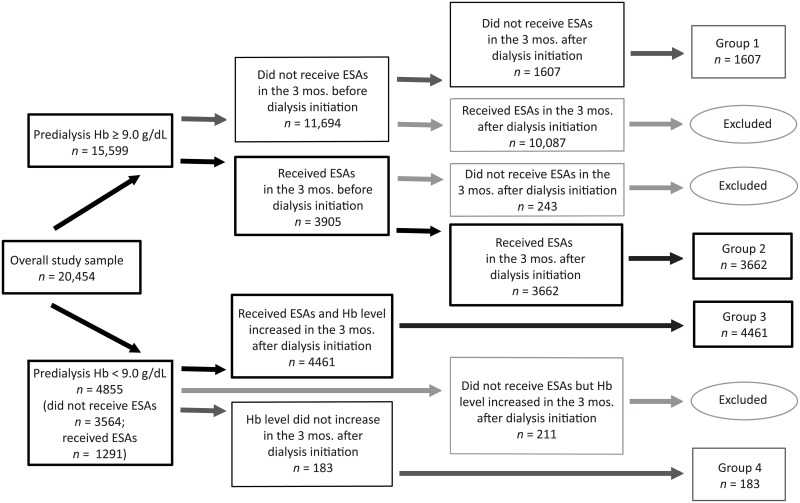
Depiction of dividing patients into four treatment groups, using Hb threshold of 9.0 g/dL.

The study concept design is shown in Fig 1, which demonstrates how patients were assigned to one of four anemia management treatment pattern groups.

#### Group 1

The first group comprised patients without anemia (defined operationally as Hb ≥ 9.0 g/dL) both upon hemodialysis initiation and afterwards and who received no ESAs in the 3 months before or in the 3 months after hemodialysis initiation. These patients presumably would never have been candidates for predialysis ESAs.

#### Group 2

The second group comprised patients who had Hb levels ≥ 9.0 g/dL while receiving ESAs both before and after hemodialysis initiation. They can be conceptualized as “maintainers,” or patients who maintained Hb ≥ 9.0 g/dL while receiving ESAs throughout the study. They comprised the comparator group.

#### Group 3

The third group comprised patients who did not attain Hb ≥ 9.0 g/dL before hemodialysis initiation (with or without ESAs), who received ESAs after hemodialysis initiation, and who experienced a subsequent increase in Hb levels after hemodialysis initiation; these patients constituted the referent group.

#### Group 4

The final group comprised patients who did not attain Hb ≥ 9.0 g/dL before hemodialysis initiation and who also did not experience an increase in Hb levels after hemodialysis initiation (presumably due to no treatment, apparent undertreatment, or ineffective treatment; this could be related to ESA resistance, but the reason cannot be known in this study); this group represented patients who did not appear to benefit from ESA treatment following hemodialysis initiation.

Because our goal was to examine the association of outcomes between two groups of patients who appeared to be fundamentally comparable except for predialysis anemia management (that is, use of ESAs and subsequent attained Hb levels), our main contrast of interest was between groups 2 and 3, although all four groups were retained for analysis. An identical analysis was undertaken using a Hb threshold of 10 mg/dL.

Additionally, we undertook a separate analysis examining outcomes in which the subgroup of patients with Hb < 9.0 g/dL who received ESAs prior to dialysis initiation were contrasted with those whose Hb levels did not increase after dialysis initiation.

### Covariates

Baseline covariates included demographic factors, primary cause of ESRD, duration of predialysis nephrology care (< 6 months, 6–12 months, > 12 months), and a broad range of comorbid conditions defined from form CMS-2728 or via International Classification of Diseases, Ninth Revision, Clinical Modification (ICD-9-CM) codes (S2 Table). A comorbid condition was defined as present by at least one inpatient, skilled nurse facility, or home health claim, or at least two outpatient or physician/supplier claims on different days.

### Outcomes

Outcomes were all-cause deaths, cardiovascular deaths, all-cause hospitalizations, and cardiovascular hospitalizations. Cause of death was obtained from the ESRD Death Notification form and is further described in S1 Text.

All-cause hospitalizations during follow-up were identified using Medicare inpatient claims. Cardiovascular hospitalizations were defined using ICD-9-CM diagnosis codes reported in the primary position or first two secondary positions of the claims (S1 Text).

### Statistical analysis

Descriptive data are displayed as percentages and means with standard deviations. Unadjusted rates of mortality and hospitalizations were calculated as total number of events divided by total time at risk (events per 100 patient-years) with 95% confidence intervals (CIs) To estimate the association between anemia management treatment patterns over the follow-up period, we used multivariate models adjusted for the covariates listed above. The modeling approach used depended on the outcome: Cox proportional hazards models for time to all-cause and cardiovascular mortality and Poisson regression models to estimate rate ratios of all-cause and cardiovascular hospitalizations.

## Results

**Fig 2:**
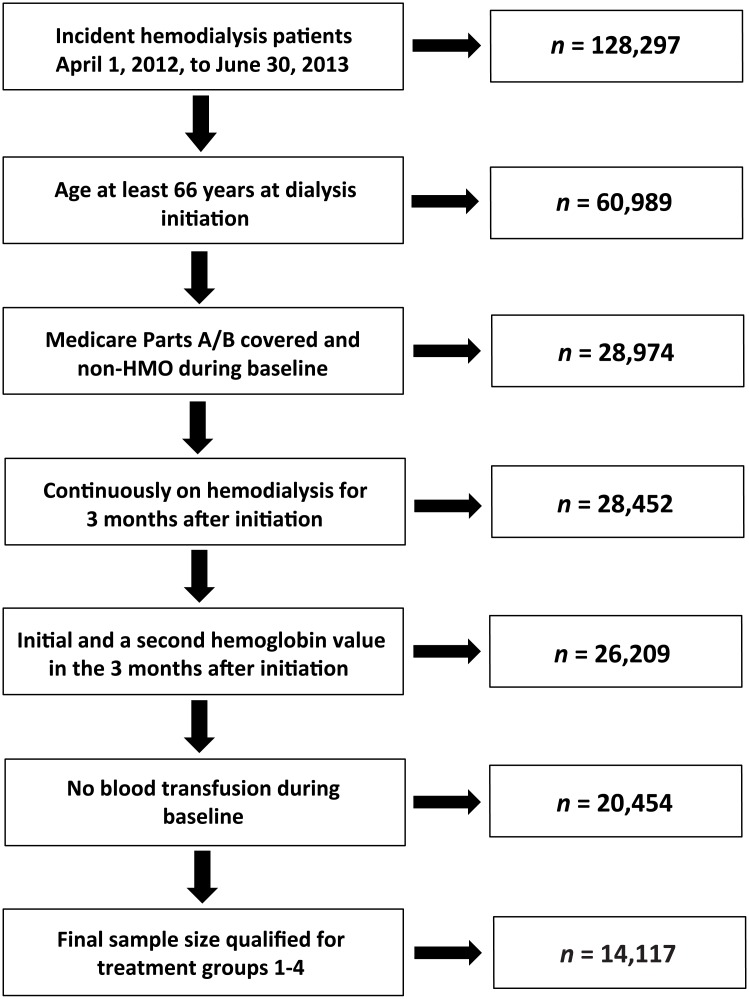
Selection of the study cohort.

Selection of the study cohort is shown in Fig 2. Within the specified study dates, 128,297 patients initiated hemodialysis. After satisfying age, insurance, and hemodialysis continuity requirements, 28,452 remained. Of these, 14,117 had adequate Hb data and had not received a blood transfusion.

Patients were next assigned to one of four groups, first using a Hb threshold of 9.0 g/dL (Fig 1) and, in a subsequent sensitivity analysis, of 10.0 g/dL (S1 Fig). Group 1 comprised the 1607 patients with predialysis Hb levels above 9.0 g/dL without ESAs. Group 2 comprised the 3662 patients who were treated with ESAs both before and after dialysis initiation and who maintained Hb levels ≥ 9.0 g/dL both before and after hemodialysis initiation. Group 3 comprised the 4461 patients whose Hb levels were < 9.0 g/dL upon hemodialysis initiation, with or without ESA treatment, but who subsequently experienced increased Hb levels while receiving ESAs after hemodialysis initiation. Group 4 comprised the 183 patients who did not attain Hb level ≥ 9.0 g/dL, even after hemodialysis initiation, with ESA treatment.

As shown in Fig 1, of the 20,454 patients initially examined, 15,599 (76.3%) had predialysis Hb levels ≥ 9.0 g/dL. Of these, only 3905 (25.0%) required ESAs to reach Hb ≥ 9.0 g/dL. Of the 4855 patients with predialysis Hb levels < 9.0 g/dL, only 1291 (26.6%) used ESAs prior to hemodialysis initiation. The 183 patients who did not experience an increase in Hb levels with ESA treatment after dialysis initiation made up only 3.8% of the predialysis anemic group; their mean Hb levels were 7.9 g/dL before hemodialysis initiation and 8.5 g/dL afterward.

**Fig 3:**
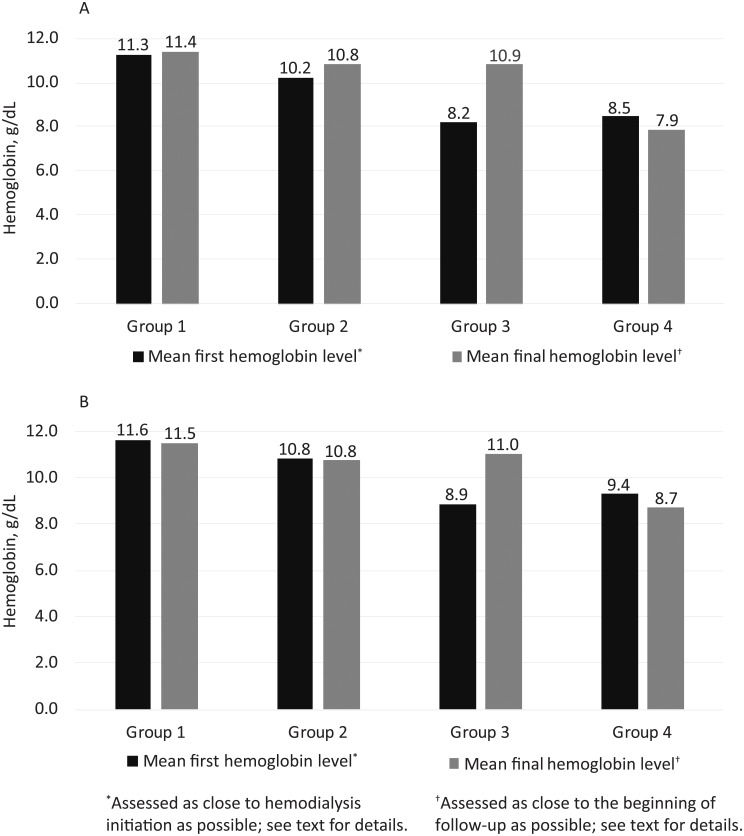
Hemoglobin changes by group, using hemoglobin thresholds of 9.0 g/dL (panel A) and 10.0 g/dL (panel B).

To confirm that our strategy was successful in differentiating these four patient groups, Hb levels by group were plotted for both Hb thresholds (Fig 3). As anticipated, patients who never needed ESAs (group 1) at any point had the highest mean Hb levels, and those whose Hb levels were ≥ 9.0g/dL with ESAs throughout (group 2) the second-highest. Patients who had Hb < 9.0 g/dL (with or without ESAs) but who received ESAs after initiation (group 3) experienced a substantial increase in mean Hb levels (from 8.2 to 10.9 g/dL for the analysis using a threshold of 9.0 g/dL, and from 8.9 to 11.0 g/dL for the analysis using a threshold of 10 g/dL). Group 4 patients consistently had the lowest Hb levels, as expected, even in the setting of ESA treatment after hemodialysis initiation.

Baseline characteristics for all groups are shown in Table 1 for the 9.0 g/dL Hb threshold and in S3 Table for 10.0 g/dL threshold. Patients in group 2, compared with those in group 3, were more likely to be older, female, and white (as compared with black); to have diabetes as cause of ESRD; to have spent less time in the hospital during the baseline period; and in general to be slightly less likely to have major comorbid conditions.

**Table 1:** Baseline characteristics and comorbidity by study group, 9.0 g/dL hemoglobin threshold.

*n*	All	Group 1	Group 2	Group 3	Group 4	*P*
	9913	1607	3662	4461	183	
Mean Hb at initiation (SD), g/dL	9.44 (1.55)	11.28 (1.31)	10.23 (0.90)	8.18 (0.80)	8.48 (0.66)	< 0.0001
Mean age (SD), yr.	76.57 (6.75)	75.91 (6.45)	77.23 (6.86)	76.28 (6.72)	75.92 (6.76)	0.0559
Age, yr.	*n*	%	%	%	%	< 0.0001
66–69	2068	22.9	18.0	22.4	24.0	
70–74	2413	25.1	23.3	24.8	26.2	
75–79	2317	22.6	24.2	23.0	23.5	
≥ 80	3115	29.4	34.5	29.8	26.2	
Sex	*n*	%	%	%	%	< 0.0001
Male	5342	70.0	49.8	51.5	52.5	
Female	4571	30.0	50.2	48.5	47.5	
Race	*n*	%	%	%	%	< 0.0001
White	7333	83.4	75.8	69.8	56.8	
Black	2054	12.9	18.6	24.5	38.8	
Other	526	3.6	5.7	5.6	4.4	
Primary cause of ESRD	*n*	%	%	%	%	0.0001
Diabetes	4545	46.6	47.7	44.2	42.6	
Hypertension	3604	34.9	36.4	36.9	33.9	
Glomerulonephritis	472	4.0	5.0	4.7	7.7	
Other	1292	14.5	11.0	14.1	15.8	
Mean total baseline hospitalization days (SD)	12.31 (17.28)	10.90 (17.71)	10.10 (14.28)	14.27 (18.84)	21.05 (20.06)	< 0.0001
Length of total baseline hospitalizations, days	*n*	%	%	%	%	< 0.0001
0	3051	38.7	34.1	25.7	19.1	
1–3	678	5.4	8.1	6.5	4.4	
> 3	6184	55.9	57.9	67.8	76.5	
Comorbid conditions	*n*	%	%	%	%	
Diabetes	7083	72.1	70.8	71.7	73.2	0.6547
ASHD	6051	63.0	60.7	60.3	68.3	0.0476
CHF	6393	65.0	63.5	64.8	72.7	0.0653
CVA/TIA	2562	22.9	25.5	27.0	29.5	0.0072
PVD	4533	41.9	45.2	47.2	54.1	0.0003
Dysrhythmia	4530	49.2	42.8	46.5	53.6	< 0.0001
Cardiac (other)	5070	51.5	49.8	51.8	59.0	0.0434
COPD	3552	38.1	33.5	36.7	42.1	0.0007
GI	1069	5.7	9.7	13.1	21.3	< 0.0001
Liver disease	815	6.3	7.9	9.0	10.9	0.0040
Cancer	1826	15.9	18.5	18.9	27.3	0.0006

ASHD, atherosclerotic heart disease; CHF, congestive heart disease; COPD, chronic obstructive pulmonary disease; CVA/TIA, cerebrovascular accident/transient ischemic attack; ESRD, end-stage renal disease; GI, gastrointestinal; Hb, hemoglobin; PVD, peripheral vascular disease.

Unadjusted rates, per 100 patient-years, of mortality and hospitalization for the 9.0 g/dL threshold are shown in S4 Table for patients in groups 2 and 3. For all outcomes assessed, namely all-cause and cardiovascular mortality and all-cause and cardiovascular hospitalization, absolute rates were higher for patients in group 3 than for patients in group 2. For example, rate of all-cause death at 3 months was 28.0 (24.8–31.2) per 100 patient-years for group 3 patients versus 20.3 (17.2–23.3) per 100 patient-years for group 2 patients; analogous rates for cardiovascular-related deaths were 11.3 (9.3–13.4) and 7.7 (5.8–9.5) per 100 patient-years. The absolute differences tended to narrow with time.

**Fig 4:**
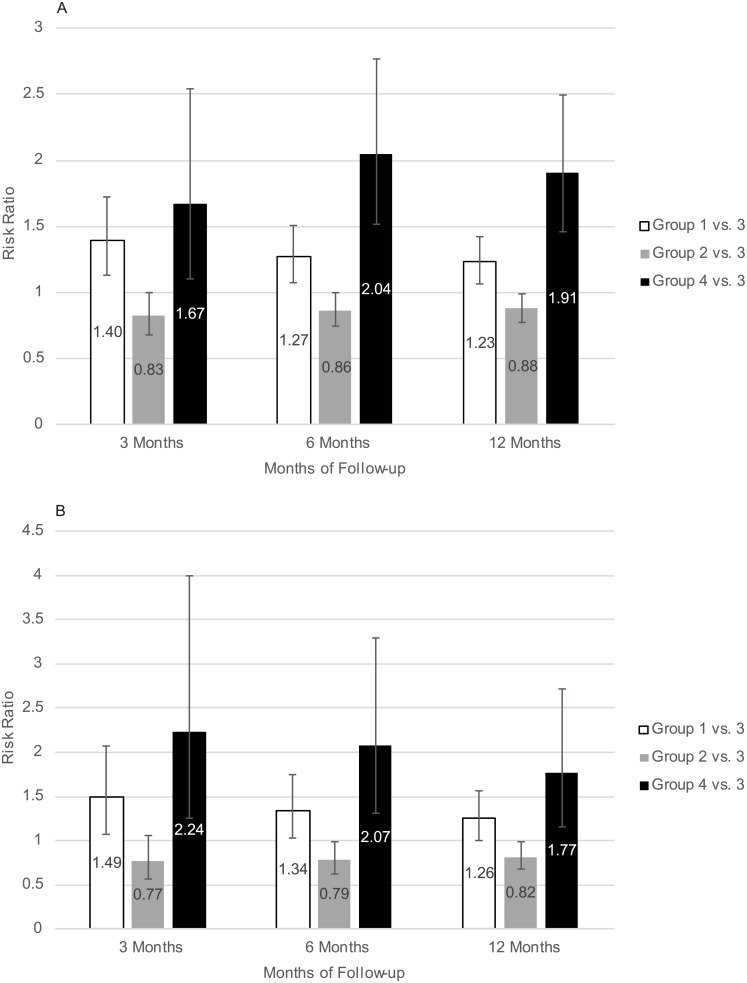
Adjusted risk of all-cause (panel A) and cardiovascular (panel B) mortality by study group over follow-up periods of 3, 6, and 12 months.

Adjusted risks of all-cause and cardiovascular mortality are shown in Fig 4. For the Hb threshold of 9.0 g/dL, all-cause mortality was less for group 2 compared with group 3 within 3 (hazard ratio [HR] 0.83, 95% CI 0.68–1.00), 6 (HR 0.86, 95% CI 0.74–1.00), and 12 (HR 0.88, 95% CI 0.78–0.99) months. The pattern was similar for cardiovascular mortality within 3 (HR 0.77, 95% CI 0.57–1.05), 6 (HR 0.79, 95% CI, 0.62–1.00), and 12 (HR 0.82, 95% CI 0.68–0.99) months. There were no significant differences between treatment groups for all-cause or cardiovascular hospitalization. The pattern of the point estimates was generally similar for the Hb threshold of 10 g/dL, but none of the risk ratios were statistically significant.

Finally, in the subgroup analysis of patients with Hb levels < 9.0 g/dL who received ESAs prior to dialysis initiation, those whose Hb levels did not increase after dialysis initiation (*n* = 1208) had a significantly higher risk of all-cause mortality at 3 (HR 2.41, 95% CI 1.09–5.34), 6 (HR 3.16, 95% CI 1.74–5.75), and 12 (HR 2.19, 95% 1.26–3.81) months compared with those whose Hb levels did increase (*n* = 50) (S5 Table); findings were similar for cardiovascular mortality (data not shown).

## Discussion

Many clinical trials and observational studies have sought to inform how ESA-based therapeutic strategies might be best used to treat anemia [11–24]. We found that in CKD patients who survived their first 90 days of dialysis, predialysis management with ESAs for those who attained a Hb level of ≥ 9.0 g/dL before hemodialysis initiation and maintained it after hemodialysis initiation was associated with a significant reduction in all-cause and cardiovascular mortality compared with Hb increases that occurred, while receiving ESA treatment, only after hemodialysis was initiated. Our findings suggest, therefore, that attainment of Hb levels ≥ 9.0 g/dL in the predialysis period while receiving ESAs, compared with non-attainment of this Hb threshold, may be associated with subsequent survival benefits. Given that care rendered in the predialysis period would likely have a diminishing effect the longer a patient lives on dialysis, the decreasing strength over time of the associations we report here was not unexpected.

Several pivotal clinic trials [12–15] demonstrated that aggressive anemia treatment targeting near normal Hb levels is not more effective than, and may be inferior to, a strategy of tolerating lower Hb levels. While these trials differed in their targeted populations (some were conducted in CKD patients [13–15] and others in hemodialysis patients [12]; some specifically targeted diabetes-related CKD [15] while others did not [12–14] and have been subject to detailed critique [25–29], their collective effect was to create substantial concern in the nephrology community about “overtreatment” of anemia in patients with non-dialysis-dependent CKD. The observation that only about one in four patients with Hb levels < 9.0 g/dL received ESAs before hemodialysis initiation is consistent with this hypothesis.

Our study was carefully designed to create a contrast between two therapeutic approaches. It differed in design from a previous study [17] that examined consistency of use in pre-hemodialysis initiation ESA dosing in that our design required the specific identification of patients who might have benefitted from a therapeutic intervention with ESAs. In patients with low predialysis Hb levels who are treated with ESAs but who do not demonstrate increased Hb levels even after dialysis initiation, predialysis use of ESAs would likely have been ineffective. Not unexpectedly, this group had the worst outcomes of the four groups; one potential explanation for this finding (in the absence of direct evidence) is that this group included the most ill and inflamed patients. In contrast, patients with (relatively) high Hb levels in the absence of ESAs both before and after dialysis initiation do not represent a true therapeutic dilemma. Outcomes for these patients were worse than for patients in the referent group (group 3), possibly because patients who initiate dialysis with relatively high Hb levels often do so primarily for indications that portend poor outcomes, such as uremic symptoms, severe hypertension, or volume overload; our study was not designed to address this issue. Our main goal, however, was specifically to study the potential effect of what might be termed a “missed treatment opportunity” to increase Hb levels before hemodialysis initiation in patients who ultimately demonstrated increased levels, while receiving ESAs, after hemodialysis initiation. While nephrologists and other providers cannot know whether an individual patient will respond to ESAs once hemodialysis is initiated, we found that only a minority of patients appeared to be unresponsive to ESAs after Hb initiation.

Our design, therefore, was primarily constructed to attempt to contrast outcomes between patients who appeared to be reasonable candidates for ESAs in the predialysis period but whose Hb did not reach 9.0 g/dL or greater (but who later appeared to be ESA-responsive after hemodialysis initiation), and those who appeared to have been more optimally managed with ESAs throughout. Differences in outcomes between these groups can therefore be seen, at least in part, as the potential cost of this putative missed treatment opportunity.

Importantly, however, our study incorporated no measure of potential responsiveness of anemia to ESAs. Providers could have had access to clinical measures of potential anemia responsiveness; such measures of putative responsiveness, to which we do not have access, would undoubtedly have informed provider decision making at the bedside. We did not seek to determine potential ESAs responsiveness in this study; rather, we sought only to determine whether any signal indicated whether non-attainment of Hb ≥ 9.0 g/dL in the predialysis setting, regardless of the cause, was associated with adverse outcomes relative to attainment of Hb ≥ 9.0 g/dL while receiving ESAs both before and after initiating dialysis.

Our study has several limitations. First, it is an observational study, and causality cannot be inferred. Second, we imposed a period of immortal time on the study sample. That is, all patients had to survive to dialysis initiation. This would introduce bias if use of ESAs were responsible for substantial mortality before dialysis initiation, in which case our design would result in a study of a cohort healthy enough to survive exposure to predialysis ESAs. However, this seems to be an unlikely threat to our findings, since the mean Hb levels in the patients who attained Hb ≥ 9.0 g/dL on ESAs throughout the study period were lower than the Hb levels associated with mortality in the CHOIR trial [14]. Third, we studied only US patients covered by Medicare, meaning that our results may not be generalizable to patients in systems of care with anemia management practices substantially different from those in the US. Fourth, we did not explicitly account for patients with recovered renal function (2% of our study sample by 1 year), principally because nephrologists and other providers likely could not have predicted recovery and, further, because most patients who eventually recovered were likely still subject to anemia management protocols involving ESAs administered by outpatient dialysis units.

Additionally, and perhaps most importantly, low Hb levels in the absence of treatment upon hemodialysis initiation likely reflect much more than a suboptimal approach to anemia management on the part of providers. Low Hb levels (either with or without ESA use) may indicate the presence of unknown patient factors that are the true source of the association we report. We attempted to at least partially control for this by incorporating the duration of predialysis nephrology care into our models.

## Conclusion

In summary, we found an association between reduced all-cause and cardiovascular mortality in patients with consistent attainment of Hb levels ≥ 9.0 g/dL while receiving ESAs compared with patients who did not attain Hb level ≥ 9.0 g/dL prior to hemodialysis initiation but who subsequently experienced an increase in Hb levels with ESA treatment once hemodialysis was initiated. Our findings suggest possible unrealized opportunities to reduce mortality in the high-risk period after dialysis initiation, but optimal management of anemia in the transition to dialysis remains uncertain.

## Supporting information

S1 Text Supplementary methods. (PDF)

S1 Table Codes used to determine use of red blood cell transfusions and erythropoiesis stimulating agents. (PDF)

S2 Table ICD-9-CM diagnosis and HCPCS codes used to identify baseline comorbid conditions. (PDF)

S3 Table Baseline characteristics for all groups, 10.0 g/dL threshold. (PDF)

S4 Table Unadjusted rates, per 100 patient-years, of mortality and hospitalization for the 9.0 g/dL threshold. (PDF)

S5 Table All-cause and cardiovascular mortality at 3, 6, and 12 months after hemodialysis initiation, patients with hemoglobin levels < 9.0 g/dL who received ESAs prior to dialysis initiation. (PDF)

S1 Fig Depiction of dividing patients into four treatment groups, using hemoglobin threshold of 10 g/dL. (PDF)

## References

[1] Freburger JK, Ng LJ, Bradbury BD, Kshirsagar AV, Brookhart MA. Changing patterns of anemia management in US hemodialysis patients. Am J Med. 2012;125: 906–914. 10.1016/j.amjmed.2012.03.011 22938926

[2] Miskulin DC, Zhou J, Tangri N, Bandeen-Roche K, Cook C, Ephraim PL, et al; DEcIDE Network Patient Outcomes in End Stage Renal Disease Study Investigators. Trends in anemia management in US hemodialysis patients 2004–2010. BMC Nephrol. 2014;14: 264. 10.1186/1471-2369-14-264 PMC3866613 24289058

[3] Fuller DS, Pisoni RL, Bieber BA, Port FK, Robinson BM. The DOPPS practice monitor for U.S. dialysis care: update on trends in anemia management 2 years into the bundle. Am J Kidney Dis. 2013;62: 1213–1216. 10.1053/j.ajkd.2013.09.006 24140369

[4] Weiner DE, Winkelmayer WC. Commentary on ‘The DOPPS practice monitor for U.S. dialysis care: update on trends in anemia management 2 years into the bundle’: iron(y) abounds 2 years later. Am J Kidney Dis. 2013;62: 1217–1220. 10.1053/j.ajkd.2013.10.006 24267389

[5] Astor BC, Muntner P, Levin A, Eustace JA, Coresh J. Association of kidney function with anemia: the Third National Health and Nutrition Examination Survey (1988–1994). Arch Intern Med. 2002;162: 1401–1408. 12076240 10.1001/archinte.162.12.1401

[6] Hsu CY, McCulloch CE, Curhan GC. Epidemiology of anemia associated with chronic renal insufficiency among adults in the United States: results from the Third National Health and Nutrition Examination Survey. J Am Soc Nephrol. 2002;13: 504–510. 11805181 10.1681/ASN.V132504

[7] Collins AJ, Li S, St.Peter WL, Ebben J, Roberts T, Ma JZ, et al Death, hospitalization, and economic associations among incident hemodialysis patients with hematocrit values of 36 to 39%. J Am Soc Nephrol. 2001;12: 2465–2473. 11675424 10.1681/ASN.V12112465

[8] Kausz AT, Solid C, Pereira BJ, Collins AJ, St Peter W. Intractable anemia among hemodialysis patients: a sign of suboptimal management or a marker of disease? Am J Kidney Dis. 2005;45: 136–147. 15696453 10.1053/j.ajkd.2004.08.042

[9] Icardi A, Paoletti E, De NL, Mazzaferro S, Russo R, Cozzolino M. Renal anaemia and EPO hyporesponsiveness associated with vitamin D deficiency: the potential role of inflammation. Nephrol Dial Transplant. 2013;28: 1672–1679. 10.1093/ndt/gft021 23468534

[10] Foley RN, Chen SC, Solid CA, Gilbertson DT, Collins AJ. Early mortality in patients starting dialysis appears to go unregistered. Kidney Int. 2014;86: 392–398. 10.1038/ki.2014.15 24522495

[11] Eschbach JW, Kelly MR, Haley NR, Abels RI, Adamson JW. Treatment of the anemia of progressive renal failure with recombinant human erythropoietin. N Engl J Med. 1989;321: 158–163. 10.1056/NEJM198907203210305 2747747

[12] Besarab A, Bolton WK, Browne JK, Egrie JC, Nissenson AR, Okamoto DM, et al The effects of normal as compared with low hematocrit values in patients with cardiac disease who are receiving hemodialysis and epoetin. N Engl J Med. 1998;339: 584–590. 10.1056/NEJM199808273390903 9718377

[13] Drueke TB, Locatelli F, Clyne N, Eckardt KU, Macdougall IC, Tsakiris D, et al; CREATE Investigators. Normalization of hemoglobin level in patients with chronic kidney disease and anemia. N Engl J Med. 2006;355: 2071–2084. 10.1056/NEJMoa062276 17108342

[14] Singh AK, Szczech L, Tang KL, Barnhart H, Sapp S, Wolfson M, et al; CHOIR Investigators. Correction of anemia with epoetin alfa in chronic kidney disease. N Engl J Med. 2006;355: 2085–2098. 10.1056/NEJMoa065485 17108343

[15] Pfeffer MA, Burdmann EA, Chen CY, Cooper ME, de Zeeuw D, Eckardt KU, et al; TREAT Investigators. A trial of Darbepoetin Alfa in type 2 diabetes and chronic kidney disease. N Engl J Med. 2009;361; 2019–2032. 10.1056/NEJMoa0907845 19880844

[16] Ma J, Ebben J, Xia H, Collins A. Hematocrit level and associated mortality in hemodialysis patients. J Am Soc Nephrol. 1999;10: 610–619. 10073612 10.1681/ASN.V103610

[17] Xue JL, St Peter WL, Ebben JP, Everson SE, Collins AJ. Anemia treatment in the pre-ESRD period and associated mortality in elderly patients. Am J Kidney Dis. 2002;40: 1153–1161. 12460033 10.1053/ajkd.2002.36861

[18] Locatelli F, Pisoni RL, Combe C, Bommer J, Andreucci VE, Piera L, et al Anaemia in haemodialysis patients of five European countries: association with morbidity and mortality in the Dialysis Outcomes and Practice Patterns Study (DOPPS). Nephrol Dial Transplant. 2004;19: 121–132. 14671047 10.1093/ndt/gfg458

[19] Bradbury BD, Wang O, Critchlow CW, Rothman KJ, Heagerty P, Keen M, et al Exploring relative mortality and epoetin alfa dose among hemodialysis patients. Am J Kidney Dis. 2008;51: 62–70. 18155534 10.1053/j.ajkd.2007.09.015

[20] Bradbury BD, Danese MD, Gleeson M, Critchlow CW. Effect of epoetin alfa dose changes on hemoglobin and mortality in hemodialysis patients with hemoglobin levels persistently below 11 g/dL. Clin J Am Soc Nephrol. 2009;4: 630–637. 10.2215/CJN.03580708 19261826 PMC2653654

[21] Winkelmayer WC. Confusion about the appropriate use of erythropoiesis-stimulating agents in patients undergoing maintenance dialysis. Semin Dial. 2010;23: 486–491. 21069924 10.1111/j.1525-139x.2010.00768.x

[22] Hahn D, Cody JD, Hodson EM. Frequency of administration of erythropoiesis-stimulating agents for the anaemia of end-stage kidney disease in dialysis patients. Cochrane Database Syst Rev. 2014;5: CD003895. 10.1002/14651858.CD003895.pub3 PMC8756398 24872328

[23] Coronado Daza J, Marti-Carvajal AJ, Ariza Garcia A, Rodelo Ceballos J, Yomayusa Gonzáles N, Páez-Canro C, et al Early versus delayed erythropoietin for the anaemia of end-stage kidney disease. Cochrane Database Syst Rev 2015;12: CD011122. 10.1002/14651858.CD011122.pub2 PMC6481893 26671531

[24] Wilhelm-Leen ER, Winkelmayer WC. Mortality risk of darbepoetin alfa versus epoetin alfa in patients with CKD: systematic review and meta-analysis. Am J Kidney Dis. 2015;66: 69–74. 25636816 10.1053/j.ajkd.2014.12.012 PMC4485593

[25] Levin A. Understanding recent haemoglobin trials in CKD: methods and lesson learned from CREATE and CHOIR. Nephrol Dial Transplant. 2007;22: 309–312. 10.1093/ndt/gfl824 17234670

[26] Berns JS, Fishbane S. CHOIR, CREATE, and anemia treatment in patients with CKD. Semin Dial. 2007;20: 277–279. 10.1111/j.1525-139X.2007.00290.x 17555496

[27] Singh AK, Fishbane S. The optimal hemoglobin in dialysis patients- a critical review. Semin Dial. 2008;21: 1–6. 10.1111/j.1525-139X.2007.00329.x 18251947

[28] Foley RN. Target hemoglobin trials in chronic kidney disease: design and interpretation issues. Pediatr Nephrol. 2009;24: 2279–2285. 10.1007/s00467-009-1123-z 19221807

[29] Teehan G, Benz RL. An update on the controversies in anemia management in chronic kidney disease: lessons learned and lost. Anemia. 2011;2011: 623673 10.1155/2011/623673 21541213 PMC3085324

