# Supplementary material for: Predialysis anemia management and outcomes following dialysis initiation: A retrospective cohort analysis
Source: PLoS One. 2018 Sep 26;13(9):e0203767. doi: 10.1371/journal.pone.0203767 (PMC6157862; doi:10.1371/journal.pone.0203767)
Supplement: S1 Text — (PDF) [file pone.0203767.s001.pdf]

S1. Text

Approach to determining hemoglobin levels in patients initiating hemodialysis

The first hemoglobin level assessed was designed to be as close to the initiation of hemodialysis as possible. If present (71.2%), the hemoglobin level on the (earliest possible) hemodialysis claim drawn from the same month as the initial dialysis date was used. If this was not present, the hemoglobin level from CROWNWeb drawn from the same month as the initial hemodialysis date was used. Finally, if the CROWNWeb value was not available, the initial hemoglobin value from the Medical Evidence Report, completed at hemodialysis initiation, was used. The first reported hemoglobin level (i.e., that obtained during the initiation month) was considered to reflect predialysis treatment (since treatments initiated immediately after hemodialysis initiation would be unlikely to have a major effect on first-month hemoglobin levels).

The second hemoglobin level, assessed from hemodialysis claims (if present, as in the majority of cases) or from CROWNWeb, was the last value during the baseline period (i.e., typically in the month prior to the initiation of the follow-up period).

Data used sources to define predialysis ESA use

(i) Medicare Parts A and B claims; detailed Healthcare Common Procedure Coding System codes for erythropoietin alfa, darbapoetin, and peginesatide are listed in **Table S1**; (ii) Medicare Part D claims, for beneficiaries, using the National Drug Codes for ESAs (**Table S1)**; (iii) the ESRD Medical Evidence Report; question 18 addresses therapy prior to ESRD, such as ESA use 6 to 12 months before initiation.

Cardiovascular causes of death

Cardiovascular disease leading to death included myocardial infarction, pericarditis including cardiac tamponade, atherosclerotic heart disease, congestive heart failure and cardiomyopathy, pulmonary edema, cardiac arrhythmia and cardiac arrest, valvular heart disease, cerebrovascular accident (ischemic or hemorrhagic), and ischemic brain damage/anoxic encephalopathy.

List of International Classification of Diseases, Ninth Revision, Clinical Modification diagnosis codes used to identify cardiovascular disease outcomes

276.6
394-398
402.01
402.11
402.91
404.01
404.03
404.11
404.13
404.91
404.93
410-414
415-424
425-428
429
430-438
