# Supplementary material for: Predialysis anemia management and outcomes following dialysis initiation: A retrospective cohort analysis
Source: PLoS One. 2018 Sep 26;13(9):e0203767. doi: 10.1371/journal.pone.0203767 (PMC6157862; doi:10.1371/journal.pone.0203767)
Supplement: S1 Table — (PDF) [file pone.0203767.s002.pdf]

Table S1. Codes used to determine use of red blood cell transfusions and erythropoiesis stimulating agents

Anemia Treatment	Codes	Sources of Claims
Transfusions	IP/SN/OP revenue: 0380, 0381, 0382, 0391<br>IP/SN/OP value: 37<br>IP/SN/OP procedure: 99.03, 99.04<br>IP/SN/OP HCPCS/CPT: P9010, P9011, P9016, P9021, P9022, P9038, P9039, P9040, P9051, P9054, P9056, P9057, P9058, 36430	Medicare Parts A/B: IP/SN/OP
EPO/DPO/PEG	EPO, HCPCS: J0885, J0886, Q4081, Q0136, Q4055; Revenue Center Code 0634 or 0635<br>DPO, HCPCS: C1774, J0880, Q4054, Q0137, J0881, J0882<br>PEG, HCPCS: Q2047, J0890	Medicare Parts A/B: IP/SN/OP
EPO/DPO/PEG	NDC codes for EPO:<br>00062030302, 00062030402, 00062031002, 00062740003, 00062740103, 00062740201, 00062740501, 54569313700, 54868252300, 54868252301, 54868542800, 54868542900, 54868567300, 54868567301, 54868580200, 54868586700, 55513000201, 55513000204, 55513000301, 55513000304, 55513000401, 55513000404, 55513000501, 55513000504, 55513000601, 55513001001, 55513001004, 55513001101, 55513001104, 55513001201, 55513001204, 55513001301, 55513001304, 55513001401, 55513001404, 55513001501, 55513002101, 55513002104, 55513002301, 55513002304 55513002501, 55513002504, 55513002701, 55513002704, 55513002801, 55513003201, 55513003701, 55513003704, 55513003901, 55513003904, 55513004101, 55513004104, 55513004301, 55513004304, 55513004401, 55513004601, 55513004801, 55513005301, 55513005304, 55513005401, 55513005404, 55513005701, 55513005704, 55513005801, 55513005804, 55513009001, 55513009101, 55513009201, 55513009301, 55513009401, 55513009501, 55513009601, 55513009701, 55513009801, 55513009804, 55513011001, 55513011101, 55513012601, 55513012610, 55513014401, 55513014410, 55513014801, 55513014810, 55513026701, 55513026710, 55513028301, 55513028310, 55513047801, 55513047810, 55513082301, 55513082310, 59676030200, 59676030201, 59676030202, 59676030300, 59676030301, 59676030302, 59676030400, 59676030401, 59676030402, 59676031000, 59676031001, 59676031002, 59676031200, 59676031201, 59676031204, 59676032000, 59676032001, 59676032004, 59676034000, 59676034001<br><br>NDC codes for DPO<br>54868542800, 54868542900, 54868586700, 55513000201, 55513000204, 55513000301, 55513000304, 55513000401, 55513000404, 55513000501, 55513000504, 55513000601, 55513001001, 55513001004, 55513001101, 55513001104, 55513001201, 55513001204, 55513001301, 55513001304, 55513001401, 55513001404, 55513001501, 55513002101, 55513002104, 55513002301, 55513002304, 55513002501, 55513002504, 55513002701, 55513002704, 55513002801, 55513003201, 55513003701, 55513003704, 55513003901, 55513003904, 55513004101, 55513004104, 55513004301, 55513004304, 55513004401, 55513004601, 55513004801, 55513005301,	Medicare Part D

	55513005304, 55513005401, 55513005404, 55513005701, 55513005704, 55513005801, 55513005804, 55513009001, 55513009101, 55513009201, 55513009301, 55513009401, 55513009501, 55513009601, 55513009701, 55513009801, 55513009804, 55513011001, 55513011101<br><br>NDC codes for PEG:<br>64764061010, 64764062020

DPO, darbepoetin alfa; EPO, erythropoietin alfa; HCPCS, Healthcare Common Procedure Coding System; IP, inpatient; NDC, National Drug Code; OP, outpatient; PEG, peginesatide; SN, skilled nursing.
