# Supplementary material for: Predialysis anemia management and outcomes following dialysis initiation: A retrospective cohort analysis
Source: PLoS One. 2018 Sep 26;13(9):e0203767. doi: 10.1371/journal.pone.0203767 (PMC6157862; doi:10.1371/journal.pone.0203767)
Supplement: S2 Table — (PDF) [file pone.0203767.s003.pdf]

Table S2. ICD-9-CM diagnosis and HCPCS codes used to identify baseline comorbid conditions

Conditions	ICD-9-CM Diagnosis and HCPCS Codes	ICD-9-CM V-Codes
ASHD	410-414	V45.81; V45.82
CHF	398.91;422; 425; 428; 402.X1; 404.x1; 404.x3	V42.1
CVA/TIA	430-438	
PVD	440-444; 447; 451-453; 557	
Dysrhythmia	426-427	V45.0; V53.3
Cardiac (other)	420-421;423-424; 429; 785.0-785.3	V42.2; V43.3
COPD	491-494; 496; 510	
GI	456.0-456.2; 530.7; 531-534; 569.84; 569.85; 578	
Liver disease	570; 571; 572.1; 572.4; 573.1-573.3	V42.7
Cancer	140-172; 174-208; 230-231; 233-234	
Diabetes	250; 357.2; 362.0x; 366.41	

ASHD, atherosclerotic heart disease; CHF, congestive heart disease; COPD, chronic obstructive pulmonary disease; CVA/TIA, cerebrovascular accident/transient ischemic attack; GI, gastrointestinal; HCPCS, Healthcare Common Procedure Coding System; ICD-9-CM, International Classification of Diseases, Ninth Revision, Clinical Modification; PVD, peripheral vascular disease.
