# Supplementary material for: Predialysis anemia management and outcomes following dialysis initiation: A retrospective cohort analysis
Source: PLoS One. 2018 Sep 26;13(9):e0203767. doi: 10.1371/journal.pone.0203767 (PMC6157862; doi:10.1371/journal.pone.0203767)
Supplement: S3 Table — (PDF) [file pone.0203767.s004.pdf]

Table S3. Baseline characteristics and comorbidity by study group, 10.0 g/dL hemoglobin threshold

	All	Group 1	Group 2	Group 3	Group 4	
n	14,117	1360	2049	9737	971	*P*
Mean hemoglobin at initiation (SD), g/dL	9.45 (1.30)	11.60 (1.15)	10.83 (0.74)	8.87 (0.86)	9.35 (0.57)	< 0.0001
Mean age (SD), yr.	76.60 (6.78)	76.02 (6.44)	77.40 (6.77)	76.54 (6.81)	76.38 (6.83)	< 0.0001
Age group	*n*	%	%	%	%	< 0.0001
66-69	2931	22.2	16.5	21.3	21.9	
70-74	3442	25.1	23.6	24.4	24.9	
75-79	3275	22.6	25.0	22.9	22.8	
≥ 80	4469	30.1	34.9	31.3	30.4	
Sex	*n*	%	%	%	%	< 0.0001
Male	7569	70.5	48.5	52.4	52.7	
Female	6548	29.5	51.5	47.6	47.3	
Race	N	%	%	%	%	< 0.0001
White	10463	84.6	76.0	72.8	68.6	
Black	2923	11.7	18.0	22.0	26.7	
Other	731	3.7	6.1	5.3	4.7	
Primary cause of ESRD	*n*	%	%	%	%	< 0.0001
Diabetes	6329	46.2	46.1	44.9	39.4	
Hypertension	5249	36.0	38.5	37.2	36.4	
Glomerulonephritis	657	3.5	4.5	4.7	5.9	
Other	1882	14.4	10.9	13.2	18.3	
Mean total baseline hospitalization days (SD)	13.09 (17.49)	10.34 (16.73)	9.75 (13.89)	13.40 (17.38)	20.93 (22.97)	< 0.0001
Length of total baseline hospitalizations, days	*n*	%	%	%	%	< 0.0001
0	3930	37.4	34.1	26.0	19.4	
1-3	949	6.0	8.4	6.7	4.2	
> 3	9238	56.5	57.4	67.3	76.4	
Comorbid conditions	*n*	%	%	%	%	
Diabetes	10096	70.8	69.2	72.0	72.3	0.0353
ASHD	8785	64.4	60.0	61.8	68.2	< 0.0001
CHF	9374	65.6	61.8	66.8	73.3	< 0.0001
CVA/TIA	3744	22.9	26.3	26.5	33.0	< 0.0001
PVD	6505	42.1	45.4	46.1	52.7	< 0.0001
Dysrhythmia	6681	50.1	42.5	47.3	54.2	< 0.0001
Cardiac (other)	7394	50.4	48.9	52.6	60.1	< 0.0001
COPD	5172	38.5	33.3	36.5	42.2	< 0.0001
GI	1582	6.0	9.5	11.6	18.0	< 0.0001
Liver disease	1187	6.5	7.8	8.4	12.5	< 0.0001
Cancer	2546	15.0	17.7	18.0	23.4	0.0005

ASHD, atherosclerotic heart disease; CHF, congestive heart disease; COPD, chronic obstructive pulmonary disease; CVA/TIA, cerebrovascular accident/transient ischemic attack; ESRD, end-stage renal disease; GI, gastrointestinal; PVD, peripheral vascular disease.
