# Supplementary material for: Predialysis anemia management and outcomes following dialysis initiation: A retrospective cohort analysis
Source: PLoS One. 2018 Sep 26;13(9):e0203767. doi: 10.1371/journal.pone.0203767 (PMC6157862; doi:10.1371/journal.pone.0203767)
Supplement: S4 Table — (PDF) [file pone.0203767.s005.pdf]

Table S4. Unadjusted rates, per 100-patient-years, of mortality and hospitalization, hemoglobin threshold 9 g/dL

Outcomes, Time after Dialysis Initiation*	Unadjusted Rate (95% CI)	
	ESA Treatment	
	Before and after Hemodialysis Initiation	Only after Hemodialysis Initiation
n	3662	4461
All-cause death		
3	20.3 (17.2-23.3)	28.0 (24.8-31.2)
6	19.4 (17.2-21.6)	25.8 (23.5-28.1)
12	18.9 (17.2-20.6)	23.8 (22.0-25.6)
CV-related death		
3	7.7 (5.8-9.5)	11.3 (9.3-13.4)
6	7.4 (6.1-8.7)	10.7 (9.2-12.2)
12	7.4 (6.3-8.4)	10.0 (8.8-11.1)
All-cause hospitalizations†		
3	172.1 (163.1-181.0)	197.0 (188.2-205.7)
6	170.2 (163.7-176.8)	186.7 (180.4-193.1)
12	163.5 (158.4-168.6)	176.8 (171.8-181.8)
CV-related hospitalizations†		
3	48.3 (43.6-53.0)	55.5 (51.0-60.1)
6	49.0 (45.6-52.5)	50.5 (47.3-53.8)
12	47.3 (44.6-50.1)	48.8 (46.2-51.4)

*Months after 90 days from dialysis initiation.

†Multiple hospitalizations.

CI, confidence interval; CV, cardiovascular; ESAs, erythropoiesis-stimulating agents.
