# Supplementary material for: Predialysis anemia management and outcomes following dialysis initiation: A retrospective cohort analysis
Source: PLoS One. 2018 Sep 26;13(9):e0203767. doi: 10.1371/journal.pone.0203767 (PMC6157862; doi:10.1371/journal.pone.0203767)
Supplement: S5 Table — (PDF) [file pone.0203767.s006.pdf]

Table S5. All-cause and cardiovascular mortality at 3, 6, and 12 months after hemodialysis initiation, patients with hemoglobin levels < 9.0 g/dL who received ESAs prior to dialysis initiation

	HR (95% CI)		
	3 Months	6 Months	12 Months
Study groups			
3A*	1.00 (reference)	1.00 (reference)	1.00 (reference)
4A†	2.41 (1.09-5.34)	3.16 (1.74-5.75)	2.19 (1.26-3.81)
Nephrologist care			
Yes	0.99 (0.59-1.66)	0.89 (0.59-1.33)	0.70 (0.51-0.98)
No	1.00 (reference)	1.00 (reference)	1.00 (reference)
Age, years			
66-69	1.00 (reference)	1.00 (reference)	1.00 (reference)
70-74	0.98 (0.47-2.07)	1.05 (0.57-1.94)	1.02 (0.62-1.70)
75-79	1.02 (0.48-2.16)	1.25 (0.69-2.29)	1.25 (0.77-2.04)
≥ 80	1.08 (0.54-2.17)	1.25 (0.70-2.22)	1.34 (0.84-2.12)
Sex			
Male	1.00 (reference)	1.00 (reference)	1.00 (reference)
Female	0.83 (0.51-1.35)	0.90 (0.61-1.32)	0.77 (0.56-1.06)
Race			
White	1.00 (reference)	1.00 (reference)	1.00 (reference)
Black	1.44 (0.83-2.49)	0.99 (0.62-1.56)	0.86 (0.59-1.26)
Other	1.11 (0.38-3.23)	0.81 (0.32-2.07)	0.69 (0.30-1.60)
Primary cause of ESRD			
Diabetes	1.00 (reference)	1.00 (reference)	1.00 (reference)
Hypertension	1.49 (0.84-2.66)	1.20 (0.75-1.91)	0.91 (0.61-1.35)
Glomerulonephritis	2.80 (1.01-7.80)	2.08 (0.89-4.87)	1.32 (0.61-2.87)
Other	1.36 (0.62-2.98)	1.22 (0.66-2.25)	1.14 (0.69-1.86)
Length of total baseline hospitalizations, days			
0	1.00 (reference)	1.00 (reference)	1.00 (reference)
1-3	2.25 (0.62-8.17)	2.19 (0.90-5.34)	1.73 (0.87-3.45)
> 3	3.23 (1.34-7.77)	2.36 (1.28-4.36)	1.99 (1.27-3.13)
Comorbid conditions			
Diabetes	1.50 (0.80-2.83)	1.41 (0.84-2.36)	1.25 (0.80-1.93)
ASHD	0.95 (0.54-1.67)	1.20 (0.76-1.91)	1.21 (0.83-1.78)
CHF	1.04 (0.55-1.95)	1.55 (0.91-2.63)	1.43 (0.93-2.20)
CVA/TIA	1.25 (0.75-2.09)	1.14 (0.76-1.73)	1.34 (0.96-1.86)
PVD	1.22 (0.73-2.04)	0.97 (0.65-1.43_	1.01 (0.73-1.39)
Dysrhythmia	1.92 (1.11-3.33)	1.47 (0.96-2.25)	1.42 (1.01-2.01)
Cardiac (other)	1.36 (0.80-2.31)	1.33 (0.87-2.04)	1.49 (1.05-2.13)
COPD	1.49 (0.91-2.43)	1.18 (0.80-1.74)	1.05 (0.76-1.45)
GI disease	1.56 (0.91-2.69)	1.56 (1.00-2.42)	1.35 (0.92-1.97)
Liver disease	2.04 (1.11-3.78)	1.53 (0.90-2.60)	1.19 (0.74-1.92)
Cancer	1.86 (1.11-3.11)	1.78 (1.17-2.71)	1.54 (1.08-2.20)

[*]Pre-hemodialysis anemia with ESA use, anemia corrected with post-hemodialysis ESA use (*n* = 1208).

[†]Pre-hemodialysis anemia with ESA use, anemia not corrected with post-hemodialysis ESA use (*n* = 50).

ASHD, atherosclerotic heart disease; CHF, congestive heart failure; CI, confidence interval; COPD, chronic obstructive pulmonary disease; CVA/TIA, cerebrovascular accident/transient ischemic attack; ESRD, end-stage renal disease; GI, gastrointestinal; HR, hazard ratio; PVD, peripheral vascular disease.
