# Supplementary material for: Predialysis anemia management and outcomes following dialysis initiation: A retrospective cohort analysis
Source: PLoS One. 2018 Sep 26;13(9):e0203767. doi: 10.1371/journal.pone.0203767 (PMC6157862; doi:10.1371/journal.pone.0203767)

Fig. S1. Depiction of dividing patients into four treatment groups, using hemoglobin threshold of 10 g/dL.

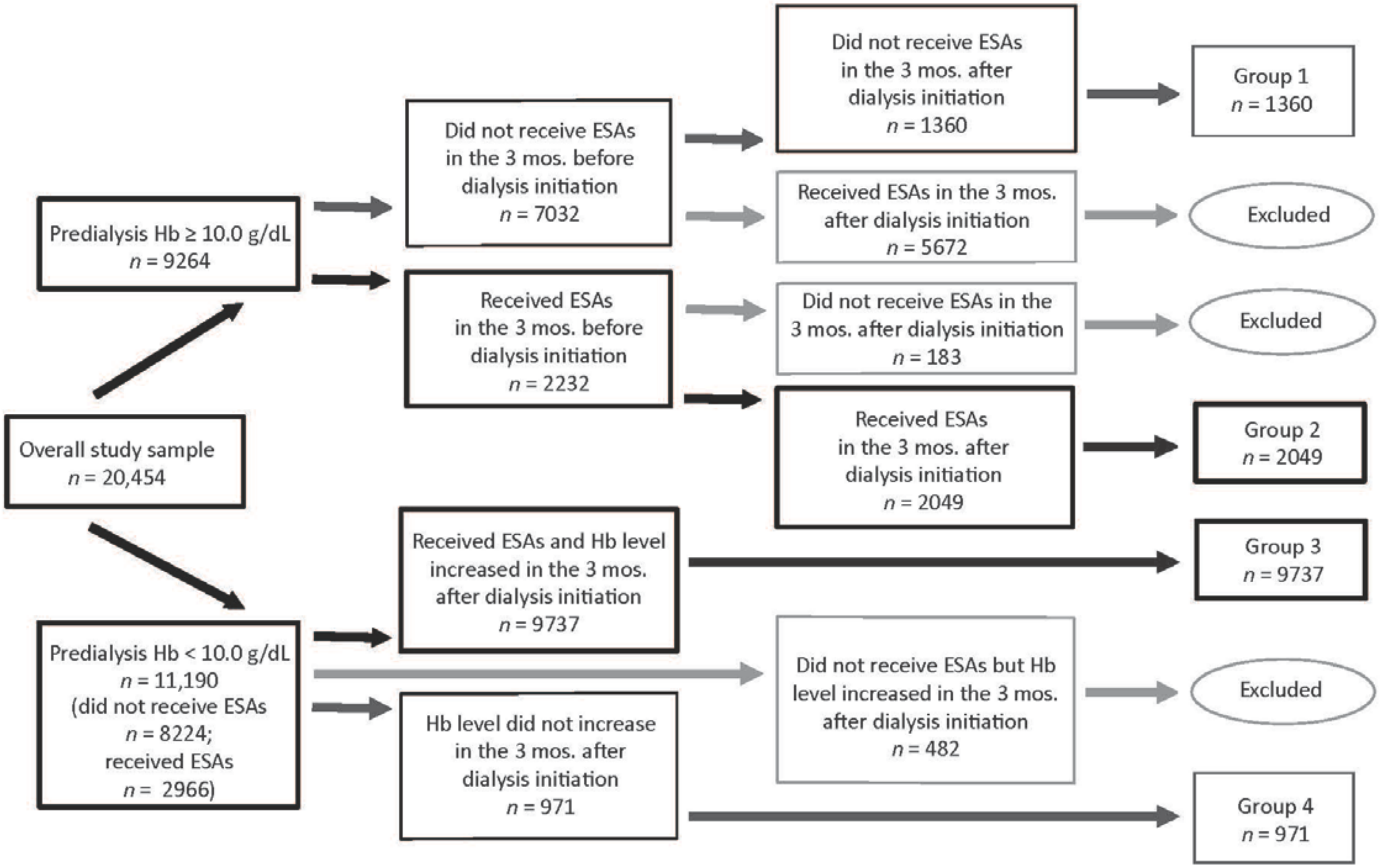

Supplement: S1 Fig — (PDF) [file pone.0203767.s007.pdf]
